# Mitogenomic Research of Silverleaf Sunflower (*Helianthus argophyllus*) and Its Interspecific Hybrids

**DOI:** 10.3390/cimb45060308

**Published:** 2023-06-02

**Authors:** Maksim S. Makarenko, Kirill V. Azarin, Vera A. Gavrilova

**Affiliations:** 1 The Laboratory of Plant Genomics, The Institute for Information Transmission Problems, 127051 Moscow, Russia; 2 The Laboratory of Molecular Genetics, Southern Federal University, 344006 Rostov-on-Don, Russia; 3 Oil and Fiber Crops Genetic Resources Department, The N.I. Vavilov All Russian Institute of Plant Genetic Resources, 190031 Saint Petersburg, Russia

**Keywords:** mitochondrial genome, *Helianthus*, hybridization, interspecific hybrids, sunflower, *H. argophyllus*

## Abstract

Interspecific hybridization is widespread for sunflowers, both in wild populations and commercial breeding. One of the most common species that can efficiently cross with *Helianthus annuus* is the Silverleaf sunflower—*Helianthus argophyllus*. The current study carried out structural and functional organization analyses of mitochondrial DNA in *H. argophyllus* and the interspecific hybrid, *H. annuus* (VIR114A line) × *H. argophyllus*. The complete mitogenome of *H. argophyllus* counts 300,843 bp, has a similar organization to the mitogenome of cultivated sunflower, and holds SNPs typical for wild sunflowers. RNA editing analysis predicted 484 sites in *H. argophyllus* mitochondrial CDS. The mitochondrial genome of the *H. annuus* × *H. argophyllus* hybrid is identical to the maternal line (VIR114A). We expected that significant rearrangements in the mitochondrial DNA of the hybrid would occur, due to the frequent recombination. However, the hybrid mitogenome lacks rearrangements, presumably due to the preservation of nuclear–cytoplasmic interaction paths.

## 1. Introduction

The genus, *Helianthus*, comprises 49–53 [1,2,3] species that occupy diverse habitats within North and South America [4]. These species possess extremely interesting genetic features, including high evolution rates, diverse ploidy levels (diploid, tetraploid, hexaploid populations/species), significant variation in genome size, even among species with the same ploidy, exceptionally high levels of genetic variation compared to other flowering plants, and substantial rates of gene flow between the species [5,6,7,8,9]. Consequently, sunflowers often serve as a model for studying genetic aspects of adaptation, hybridization, and speciation [10,11,12]. Additionally, the cultivated sunflower (*Helianthus annuus* L.) is one of the world’s major food crops, making research on sunflowers valuable from both theoretical and practical perspectives.

The cultivated sunflower has a narrow genetic base due to its recent origin, domestication, and breeding [13]. However, wild sunflower species have adapted to a wide range of environments during their dispersion and possess considerable genetic variability that could be utilized to improve sunflower resistance to biotic and abiotic stresses [3,14]. Numerous resistance genes for major diseases, such as rust, downy mildew, wilt, stem canker, black stem, have been reported in the wild sunflower species [3,13,15]. Broadening the genetic base of cultivated sunflowers is often achieved through introgressive hybridization with wild species [16]. The transfer of beneficial traits from *Helianthus* species to cultivated sunflowers has been ongoing since the early 20th century, with current efforts focused on accelerating this process [17].

One potential donor species for beneficial alleles is the Silverleaf sunflower (*H. argophyllus* Torrey and Gray), a weed plant that inhabits sandy soils in south and southeast Texas [18]. *H. argophyllus* has been extensively used as a source of advantageous alleles for disease resistance [19], as well as for salt and drought tolerance [20], and insect resistance [12,21]. Comparative analysis has shown that hybrids of cultivated sunflowers with wild cytoplasmic male sterility (CMS) sources (*H. argophyllus*, *H. debilis*, *H. praecox*) exhibit a positive heterosis effect on seed yield and oil content [22]. *H. annuus* and *H. argophyllus* are closely related species with common morphological features [23], and natural hybrids between the two have been found in Texas [4]. Gene flow events occur between *H. annuus* and *H. argophyllus* species [5]. Thus, the crosses between these sister species have usually resulted in fertile progeny [24].

Mitochondrial DNA (mtDNA) is susceptible to frequent recombination, leading to significant structural changes (rearrangements) [25,26]. However, the rates of mtDNA recombination are strictly controlled by nuclear genes [27,28]. Hybridization, in turn, results in heterogeneity of the nuclear genetic apparatus in a hybrid [29], disrupting the balance of nuclear–cytoplasmic interactions and potentially causing cytonuclear conflicts that affect the frequency of mitochondrial genome rearrangements [30,31]. Unfortunately, few studies have investigated the role of mitochondrial genome variability in interspecific hybrids of higher plants, and to the best of our knowledge, such studies are lacking in sunflowers. Furthermore, interspecific hybridization is one of the ways to develop new cytoplasmic male sterility sources [32,33,34]. Additionally, mitogenome investigations in interspecific hybrids may help to elucidate the mechanisms of CMS occurrence.

The current study aimed to analyze the structural and functional organization of mtDNA in *H. argophyllus*, and the interspecific hybrid, *H. annuus* × *H. argophyllus*. We examined the mitochondrial gene profile and editing patterns for *H. argophyllus* and the hybrid.

## 2. Materials and Methods

### 2.1. Plant Material, Mitochondrial DNA Extraction

Three sunflower samples, namely *H. annuus* VIR114A line (CMS line with PET1 source), *H. argophyllus* 1000, and the F1 hybrid—*H. annuus* VIR114A × *H. argophyllus* 1000—were obtained from the genetic collection of the N. I. Vavilov All-Russian Institute of Plant Genetic Resources. Plant leaves of 21-day-old seedlings were used for mitochondrial DNA isolation. For this purpose, 10 g of leaves (without petiole and midrib) from 10 plants (1 g from each plant) were homogenized by mortar and pestle in 50 mL of STE buffer (0.4 M sucrose, 50 mM Tris pH 7.8, 4 mM EDTA-Na2, 0.2% bovine serum albumin, 0.2% 2-mercaptoethanol), filtered twice with 100-micron mesh and then centrifuged using several steps: (1) 200× *g* for 5 min, picking the supernatant; (2) 3500× *g* for 5 min, picking the supernatant; (3) 4000× *g* for 5 min, picking the supernatant; (4) 12,000× *g* for 10 min, discarding the supernatant. The pellet was treated using 10 units of DNAse (Syntol, Moscow, Russia) for 5 min and then used for DNA isolation. For both samples, the DNA extraction was performed with the PhytoSorb kit (Syntol, Moscow, Russia), according to the manufacturer’s protocol. The DNA concentration was measured with a Qubit 4 fluorometer (Thermo Fisher Scientific, Waltham, MA, USA).

### 2.2. Next-Generation Sequencing

In case of VIR114A line and the hybrid (VIR114A × *H. argophyllus*), 40–50 ng of extracted DNA was fragmented using a Covaris S220 sonicator (Covaris, Woburn, MA, USA). Then, NGS libraries were prepared with NEBNext Ultra II DNA Library Prep Kit for Illumina (New England Biolabs, Ipswich, MA, USA), following the manufacturer’s guidelines and using 10 PCR cycles. The NGS library for *H. argophyllus* was made with Nextera XT DNA Library Prep Kit (Illumina, San Diego, CA, USA), following the guidelines of Illumina.

The fragment length distribution of the prepared libraries was determined with Bioanalyzer 2100 (Agilent, Santa Clara, CA, USA), and the concentrations were evaluated with Qubit 4 fluorometer (Thermo Fisher Scientific, Waltham, MA, USA) and qPCR.

The NGS libraries of VIR114A line and the hybrid were sequenced on MiSeq (Illumina, San Diego, CA, USA) with MiSeq Reagent Kit v2 (500 cycles), while *H. argophyllus* library was sequenced on NextSeq 500 (Illumina, San Diego, CA, USA) with Mid Output Kit v2.5 (150 cycles). The following NGS data were obtained: *H. argophyllus*—4.2 million paired reads (76 + 68 bp), VIR114A—0.8 million paired reads (251 + 251 bp), VIR114A × *H. argophyllus* hybrid—8.4 million paired reads (251 + 251 bp).

### 2.3. Mitochondrial Genome Assembly and Annotation

For quality control of reads, we used FastQC v0.11.9 “https://www.bioinformatics.babraham.ac.uk/projects/fastqc/ (accessed on 31 May 2023)”. Trimmomatic v0.39 software [35] with several options (ILLUMINACLIP, SLIDINGWINDOW:5:15, MINLEN:65) was used to trim adapters and discard short or low-quality reads. *H. argophyllus* and the hybrid contigs were generated with SPAdes Genome Assembler v3.13.1 [36] using 65 k-mer. The whole mitochondrial genome assemblies were based on high coverage (>50 depth) contigs, selected using Bandage v0.8.1 [37] program for visualizing de novo assembly graphs. The genome assemblies were validated by remapping reads with Bowtie 2 v2.3.5.1 [38] and visual revision of coverage uniformity (especially in the junctions of contigs) using Tablet v1.19.09.03 [39]. For VIR114A, there was no sufficient sequencing data for de novo assembly and only mapping of reads to the reference mitogenome (MG735191.1) was provided. The mitochondrial genomes were annotated with GeSeq [40] and ORFfinder “https://www.ncbi.nlm.nih.gov/orffinder (accessed on 31 May 2023)”. The whole-genome alignments were performed with Mauve tool v2.4.0 [41].

### 2.4. SNV Localisation and RNA Editing Analysis

Bowtie 2 v2.3.5.1 was used to map reads. Sorted files in bam format were obtained with samtools v 1.16 [42]. Then, SNP calling was conducted using GATK software v 4.1.4.1 “https://gatk.broadinstitute.org (accessed on 31 May 2023)”. RNA editing events were predicted on the online website, PREPACT3 “http://www.prepact.de (accessed on 31 May 2023)” [43] using *Arabidopsis thaliana* CDS/protein database and standard option values for the prediction. Additionally, we analyzed RNA editing sites by mapping RNA-seq reads using another method. We used SRA data (SRR11923009, SRR11923010, SRR11923011, SRR11923012, SRR11923013), which were obtained from the total RNA of *H. annuus* developing seeds. First, reads obtained from NCBI SRA were mapped using hisat2 [44]. Then, the variant calling was provided with bcftools 1.9, and the RNA editing profile was analyzed with the REDO tool v 1.0 [45]. We performed manual curation using genomic viewer Tablet v 1.19.09.03 [39] to revise RNA editing results (mapped reads).

## 3. Results

**Figure 1:**
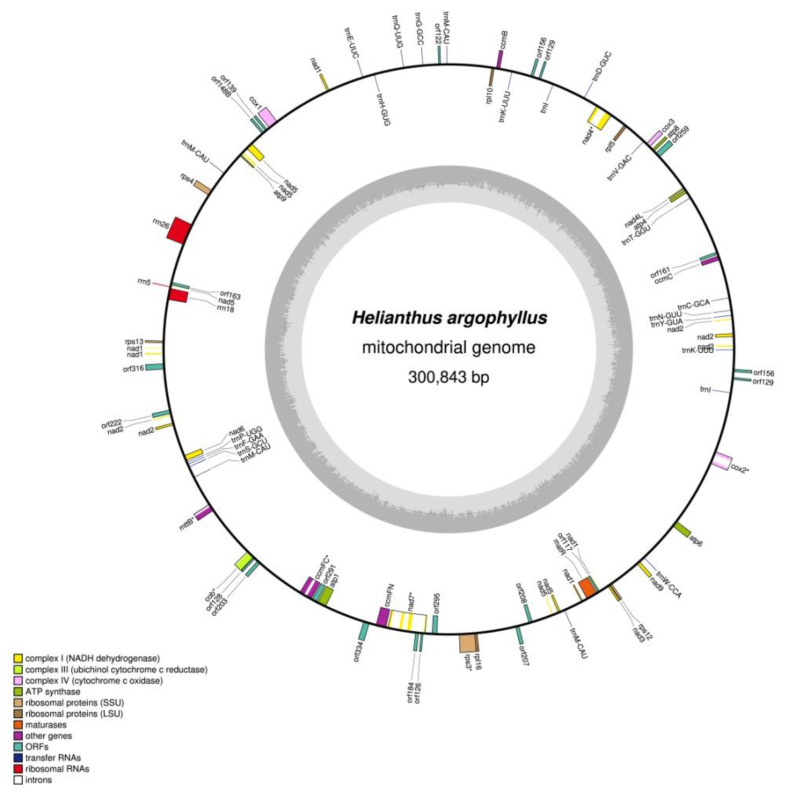
Mitochondrial genome map of *H. argophyllus.* Genes and ORFs are showed on the map and colored according their function. Intron-containing genes are marked by an asterisk (*) symbol.

The complete mitogenomes of *H. argophyllus* and the VIR114A × *H. argophyllus* hybrid were assembled de novo. In the case of the maternal CMS line (VIR114A), we performed read mapping on the reference genome (MG735191.1 NCBI accession) of another line with the same CMS type (PET1). The *H. argophyllus* and VIR114A × *H. argophyllus* assembly graphs presented in Figure S1 suggest that the mitogenomes can have different conformations (e.g., subcircles). Unfortunately, we did not have long reads to accurately evaluate the mitogenome structure isoforms. Therefore, we focused on the master cycle mitochondrial chromosome structure, confirmed by PCR and reads remapping. The master cycle mitochondrial chromosomes of both samples were assembled, and the *H. argophyllus* mitogenome was found to be 300,843 bp (Figure 1), while that of the VIR114A × *H. argophyllus* hybrid was 305,218 bp. Both mitogenomes had the same GC content—45.04%.The mitogenome of *H. argophyllus* was 104 bp shorter than the mitochondrial DNA of *H. annuus* fertile line HA89 (MN171345.1 NCBI accession) and does not have vast rearrangements (Figure S2). However, we identified several polymorphic sites (Table S1).

We then established that the VIR114A × *H. argophyllus* hybrid mitochondrial DNA was identical to the maternal (VIR114A) line and had only 2 SNP compared with PET1 reference mitogenome (MG735191.1 NCBI accession). Of note, a single nonsynonymous mutation in the *atp6* gene (Table S1) was found to be common to the hybrid and its maternal and parental forms, as well as all wild sunflowers whose mitogenomes have been described earlier [46,47] and are available in the NCBI database, including *H. grosseserratus* (MT588180.1), *H. occidentalis* (MZ147621.1), *H. strumosus* (MT588181.1), and *H. tuberosus* (MZ147622.1). However, this mutation was absent from the available mitogenomic data of sunflower cultivars (SF193, HA89, HA89(PET1), HA412).

We evaluated the gene content and predicted RNA editing sites in the mitochondrial genomes of the investigated samples. The mitochondrial genes content was the same in all studied samples, counting 31 protein-coding genes, 3 rRNA, 22 tRNA genes, and several open reading frames (ORFs). In the studied mitogenomes we annotated protein-coding genes typical for plant mitogenomes, including: nine NADH:ubiquinone oxidoreductase genes (*nad1*, *-2*, *-3*, *-4*, *-4L*, *-5*, *-6*, *-7*, *-9*), three cytochrome c oxidase genes (*cox1*, *-2*, *-3*), five ATP synthase genes (*atp1*, *-4*, *-6*, *-8*, *-9*), four cytochrome c biogenesis genes (*ccmB*, *-C*, *-FC*, *-FN*), seven ribosomal protein genes (*rps3*, *-4*, *-12*, *-13*, *rpl-5*, *-10*, *-16*), *cob*, *matR*, *mttB*. In the mitogenomes of *H. argophyllus* and the VIR114A × *H. argophyllus* hybrid, we located 22 ORFs (20 unique and 2 duplicated). Among 20 ORFs, the 2 samples shared 19 in common, while 1 ORF is different. The hybrid has a PET1 CMS-specific ORF (orfH522), which is absent in *H. argophyllus.* Conversely, the *H. argophyllus* mitogenome contains orf203, which is lacking in the hybrid.

**Figure 2:**
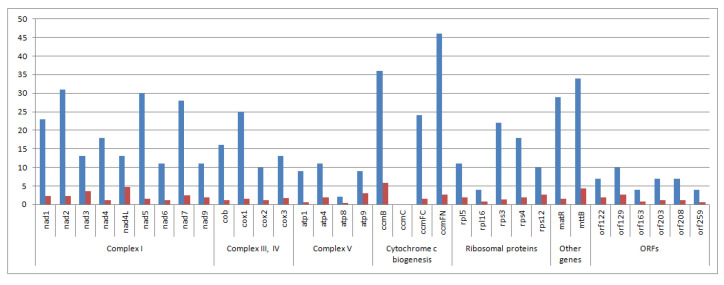
The absolute number of RNA editing substitutions per gene (blue bars) and the relative number of RNA editing substitutions by gene length normalized to 100 bp (red bars) revealed with the prediction method.

The RNA editing analysis (prediction based on *Arabidopsis thaliana* data) indicated a total of 523 RNA editing sites in the *H. argophyllus* mitogenome, with 484 in common genes and the remaining 39 in several ORFs (Figure 2). Since the sequence of the hybrid mitochondrial DNA is mainly similar to *H. argophyllus*, the result of the RNA editing prediction was almost the same except for orf203 (7 editing sites), which was absent in the hybrid.

The RNA editing analysis provided by mapping RNA-seq reads revealed 445 editing sites in common mitochondrial genes (Table S2, Figure S3). Both methods (prediction and reads mapping) showed similar results. For instance, the *nad9*, *atp9*, *rps12*, *cox3* genes had the same editing profile. Most genes had differences in 1–3 editing sites. However, we discovered a significant difference (>8 editing sites) in *ccmB* and *ccmFN* genes. Therefore, some prediction results were not supported by SRA reads mapping. These differences may be associated with tissue-specific RNA sites. Additionally, in the case of RNA-seq reads mapping, synonymous RNA editing sites were detected, while the prediction technique did not reveal such positions.

Editing sites were found in all protein-coding mitochondrial transcripts except the *atp6* gene. RNA prediction was not performed for *rps13* and rpl10 since these genes are absent in *Arabidopsis thaliana.* However, in the case of reads mapping there were 4 and 5 editing sites located for *rps13* and *rpl10*, respectively. Predominantly, the ATPase subunits, ribosomal proteins (*rpl5*, *rpl16*, *rps12*), and ORFs have a small number of RNA editing-derived substitutions. In contrast, the transcripts of NADH dehydrogenase subunits (*nad1*, nad2, *nad5*, *nad7*), cytochrome c biogenesis genes (*ccmB*, *ccmC*, *ccmFC*, *ccmFN*), *cox1*, *matR*, and *mttB* have been significantly edited (20–39 sites; Figure 2). When normalizing gene length (100 bp), we discovered a slightly different editing profile. For example, the most edited genes (>3.9 editing sites per 100 bp of gene length) were the following: *nad3*, *nad4L*, *ccmB*, *ccmC*, *mttB* (Figure 2). The absolute number of RNA editing sites is usually presented in studies [48,49,50]. However, the gene size significantly differs between mitochondrial genes. We suppose that taking into account the genes size (relative number of RNA editing events) shows more precise results.

## 4. Discussion

*H. argophyllus* is the second annual species of the *Helianthus* genus with an assembled mitogenome. According to the current data, both annual species (*H. annuus*, *H. argophyllus*) have similar mitochondrial genome organization, differing from those described in the perennial sunflower species (*H. grosseserratus*, *H. occidentalis*, *H. strumosus*, *H. tuberosus*) [46,47]. The most notable difference is that annual species have a long repeat region (about 12.9 kbp), which is absent or significantly reduced in perennial species [46,47]. The non-tandem repeats are important for mitochondrial DNA maintenance since they play a role in recombination [51]. Thus, the difference may be essential from an evolutionary point of view.

The *H. annuus* (VIR114A) × *H. argophyllus* hybrid has a maternal pattern of mitochondrial DNA inheritance. Quite unexpectedly, we found no changes in mitochondrion structure in the interspecific hybrid. In natural populations, between wild forms of *H. annuus* and *H. argophyllus*, gene drift, introgression, and hybridization are often observed [4,5,52,53], indicating a relatively weak species divergence. However, if two species do not have crucial differences (structural rearrangements of chromosomes, aneuploidy, etc.) in the nuclear genome, there is a possibility that intracellular regulation was not significantly disturbed during their hybridization. In turn, nuclear–cytoplasmic interactions may not undergo tremendous changes, and the recombination activity of the mitochondrial genome will be under active control. Of course, this is only an assumption. However, if future studies mention such a trend, there should be some modifications in breeding programs. For instance, investigations concentrating on obtaining diverse CMS sources from hybrids of closely related species, such as *H. annuus* and *H. argophyllus* [54], may have failed results.

A limited number of investigations are devoted to the alterations in mitochondrial genomes during interspecific or intraspecific hybridization. For example, the hybrids of *Solanum* species (*S. stoloniferum* and *S. tuberosum*) had rearrangements in mitochondrial DNA. In the case of cucumber (*Cucumis sativus*) hybrids, there is a transmission of mitogenome inheritance patterns (from maternal to paternal).

## 5. Conclusions

In the current study, we first assembled and described the mitogenomes of *H. argophyllus* and the interspecific hybrid, *H. annuus* (VIR114A line) × *H. argophyllus*. The *H. argophyllus* mitochondrial genome structure and gene content are similar to fertile lines of *H. annuus* (e.g., HA89). At the same time, the hybrid has a maternal (VIR114A line) inheritance of mitogenome, which, in turn, is almost identical to the mitochondrion of the line with a PET1 CMS source (HA89(PET1)). The prediction of RNA editing allowed the identification of 523 sites and the most edited genes, which belong to NADH dehydrogenase subunits and cytochrome c biogenesis genes. The data obtained in the current research could be useful for future investigations associated with CMS breeding and studies of plant mitochondrial DNA organization.

## Data Availability

The complete mitochondrial genome of *H. argophyllus* was deposited to NCBI with following GenBank accession number OQ319153. The NGS reads were deposited to NCBI SRA with the following accessions *H. argophyllus*—SRR24422231, *H. annuus* VIR114A × *H. argophyllus*—SRR23457292 and SRR23457293.

## Supplementary Materials

The following supporting information can be downloaded at: https://www.mdpi.com/article/10.3390/cimb45060308/s1, Figure S1: manually curated final assembly graphs of (A) *H. argophyllus* and (B) *H. annuus* VIR114A × *H. argophyllus* hybrid, visualized using Bandage software. Figure S2: the progressive MAUVE alignment of complete mitogenomes—*H. annuus* fertile line HA89 (MG735191.1 NCBI accession), *H. argophyllus,* and the hybrid (*H. annuus* VIR114A × *H. argophyllus*). Figure S3: the absolute number of RNA editing substitutions per gene (blue bars) and the relative number of RNA editing substitutions by gene length, normalized to 100 bp (red bars), revealed by RNAseq reads mapping. Table S1: polymorphic sites localized in *H. argophyllus* and the hybrid (*H. annuus* VIR114A × *H. argophyllus*) mitogenomes while comparing with the *H. annuus* fertile line HA89 (MG735191.1 NCBI accession). Table S2: manually revised REDO results (RNA editing sites) revealed by RNA-seq reads mapping.
