# Supplementary material for: Mitogenomic Research of Silverleaf Sunflower (Helianthus argophyllus) and Its Interspecific Hybrids
Source: Curr Issues Mol Biol. 2023 Jun 2;45(6):4841–9. doi: 10.3390/cimb45060308 (PMC10297576; doi:10.3390/cimb45060308)
Supplement: Supplementary file 1 [file cimb-45-00308-s001.zip › cimb-2329905-supplementary.pdf]

Table S1. Polymorphic sites localized in *H. argophyllus* and the hybrid (*H. annuus* VIR114A x *H. argophyllus)* mitogenomes while comparing with *H. annuus* fertile line HA89 (MG735191.1 NCBI accession)

HA89 line mitogenome position	HA89 line sequence	*H. argophyllus* mitogenome position	*H. argophyllus* sequence	The hybrid mitogenome position	The hybrid sequence	Localization/ mutation
36361	T	36359	T	36360	**G**	orf259/ Asp251Glu
36540	G	36539	**T**	36539	G	IGR*
37267	G	37266	**A**	37266	G	IGR
75333	A	75332	A	75328	**C**	IGR
116778	G	116762	G	116773	T	IGR
133548	T	133440	**A**	133543	T	IGR
133550	T	133442	**G**	133545	T	IGR
133551	A	133443	**G**	133546	A	IGR
147359	C	147252	**A**	147354	C	IGR
169030	G	168923	G	169025	**T**	nad6/ Ser232Tyr
190815	G	190712	G	202153	A	IGR
211811	**C**	211708	G	216085	G	IGR
230114	A	230011	A	234388	**C**	rpl16/ Lys32Gln
269064	**G**	268958	C	273334	C	atp6/ Lys46Asn
293547	T	293441	**G**	297818	T	IGR

*IGR – intergenic regions

Table S2. Manually revised REDO results (RNA editing sites) revealed by RNA-seq reads mapping. RNA editing sites, the same as obtained by the prediction method, are in bold

Gene name	Gene position	Amino acid position	Position in codon	Substitution type (Ref->Alt)	Substitution in codon (Ref->Alt)	Amino acid substitution (Ref->Alt)
atp1	**1490**	**497**	**2**	**C->T**	**CCA->CTA**	**P->L**
	**1292**	**431**	**2**	**C->T**	**CCG->CTG**	**P->L**
	**1216**	**406**	**1**	**C->T**	**CTT->TTT**	**L->F**
	**1178**	**393**	**2**	**C->T**	**TCA->TTA**	**S->L**
	**1064**	**355**	**2**	**C->T**	**TCG->TTG**	**S->L**
	**1039**	**347**	**1**	**C->T**	**CCC->TCC**	**P->S**
atp4	**407**	**136**	**2**	**C->T**	**CCA->CTA**	**P->L**
	**395**	**132**	**2**	**C->T**	**TCA->TTA**	**S->L**
	251	84	2	C->T	CCG->CTG	P->L
	248	83	2	C->T	CCT->CTG	P->L
	**215**	**72**	**2**	**C->T**	**TCA->TTA**	**S->L**
	**138**	**46**	**3**	**C->T**	**ATC->ATT**	**I->I**
	**118**	**40**	**1**	**C->T**	**CGT->TGT**	**R->C**
	**89**	**30**	**2**	**C->T**	**TCA->TTA**	**S->L**
	**71**	**24**	**2**	**C->T**	**TCA->TTA**	**S->L**
	**59**	**20**	**2**	**C->T**	**TCT->TTT**	**S->F**
atp8	**47**	**16**	**2**	**C->T**	**TCA->TTA**	**S->L**
	58	20	1	C->T	CTC->TTC	L->F
	**452**	**151**	**2**	**C->T**	**CCA->CTA**	**P->L**

atp9	**262**	**88**	**1**	**C->T**	**CGA->TGA**	**R->***
	**254**	**85**	**2**	**C->T**	**TCC->TTC**	**S->F**
	**251**	**84**	**2**	**C->T**	**TCA->TTA**	**S->L**
	**230**	**77**	**2**	**C->T**	**CCA->CTA**	**P->L**
	**221**	**74**	**2**	**C->T**	**TCG->TTG**	**S->L**
	**173**	**58**	**2**	**C->T**	**TCA->TTA**	**S->L**
	**131**	**44**	**2**	**C->T**	**TCG->TTG**	**S->L**
	**121**	**41**	**2**	**C->T**	**CTT->TTT**	**L->F**
	**89**	**30**	**2**	**C->T**	**TCA->TTA**	**S->L**
ccmB	**43**	**15**	**1**	**C->T**	**CCC->TCC**	**P->S**
	**68**	**23**	**2**	**C->T**	**TCT->TTT**	**S->F**
	**71**	**24**	**2**	**C->T**	**CCA->CTA**	**P->L**
	**80**	**27**	**2**	**C->T**	**TCA->TTA**	**S->L**
	**128**	**43**	**2**	**C->T**	**TCA->TTA**	**S->L**
	**137**	**46**	**2**	**C->T**	**TCC->TTC**	**S->F**
	188	63	2	C->T	CCT->CTT	P->L
	**193**	**65**	**1**	**C->T**	**CCT->TCT**	**P->S**
	**286**	**96**	**1**	**C->T**	**CGG->TGG**	**R->W**
	**304**	**102**	**1**	**C->T**	**CGT->TGT**	**R->C**
	**313**	**105**	**1**	**C->T**	**CGT->TGT**	**R->C**
	**338**	**113**	**2**	**C->T**	**CCG->CTG**	**P->L**
	**367**	**123**	**1**	**C->T**	**CGG->TGG**	**R->W**
	**380**	**127**	**2**	**C->T**	**CCG->CTG**	**P->L**
	**424**	**142**	**1**	**C->T**	**CGT->TGT**	**R->C**
	**428**	**143**	**2**	**C->T**	**TCG->TTG**	**S->L**
	**467**	**156**	**2**	**C->T**	**TCG->TTG**	**S->L**
	**503**	**168**	**2**	**C->T**	**CCA->CTA**	**P->L**
	512	171	2	C->T	TCT->TTT	S->F
	514	172	1	C->T	CGT->TGT	R->C
	**551**	**184**	**2**	**C->T**	**TCA->TTA**	**S->L**
	**554**	**185**	**2**	**C->T**	**TCG->TTG**	**S->L**
	**572**	**191**	**2**	**C->T**	**CCG->CTG**	**P->L**
	**596**	**199**	**2**	**C->T**	**TCG->TTG**	**S->L**
	**611**	**204**	**2**	**C->T**	**TCA->TTA**	**S->L**
ccmC	**673**	**225**	**1**	**C->T**	**CCT->TCT**	**P->S**
	**656**	**219**	**2**	**C->T**	**CCA->CTA**	**P->L**
	**619**	**207**	**1**	**C->T**	**CGT->TGT**	**R->C**
	**614**	**205**	**2**	**C->T**	**TCA->TTA**	**S->L**
	**608**	**203**	**2**	**C->T**	**CCC->CTC**	**P->L**
	**605**	**202**	**2**	**C->T**	**TCC->TTC**	**S->F**
	**575**	**192**	**2**	**C->T**	**CCC->CTC**	**P->L**
	**568**	**190**	**1**	**C->T**	**CCT->TCT**	**P->S**
	**521**	**174**	**2**	**C->T**	**TCG->TTG**	**S->L**
	**497**	**166**	**2**	**C->T**	**TCT->TTT**	**S->F**
	436	146	1	C->T	CCT->TCT	P->S
	**421**	**141**	**1**	**C->T**	**CGT->TGT**	**R->C**
	**400**	**134**	**1**	**C->T**	**CTT->TTT**	**L->F**
	**395**	**132**	**2**	**C->T**	**TCG->TTG**	**S->L**
	**331**	**111**	**1**	**C->T**	**CGG->TGG**	**R->W**
	**299**	**100**	**2**	**C->T**	**TCT->TTT**	**S->F**

	**281**	**94**	**2**	**C->T**	**ACA->ATA**	**T->I**
	**184**	**62**	**1**	**C->T**	**CGG->TGG**	**R->W**
	**179**	**60**	**2**	**C->T**	**GCG->GTG**	**A->V**
	**161**	**54**	**2**	**C->T**	**CCT->CTT**	**P->L**
	**133**	**45**	**1**	**C->T**	**CTT->TTT**	**L->F**
	115	39	1	C->T	CGG->TGG	R->W
	**103**	**35**	**1**	**C->T**	**CAT->TAT**	**H->Y**
	**76**	**26**	**1**	**C->T**	**CGG->TGG**	**R->W**
	5	2	2	C->T	TCC->TTC	S->F
ccmFC	**1615**	**539**	**1**	**C->T**	**CGA->TGA**	**R->***
	**1568**	**523**	**2**	**C->T**	**TCG->TTG**	**S->L**
	1539	513	3	C->T	ATC->ATT	I->I
	**1534**	**512**	**1**	**C->T**	**CGG->TGG**	**R->W**
	1439	487	2	C->T	CCG->CTG	P->L
	**1439**	**480**	**2**	**C->T**	**CCA->CTA**	**P->L**
	1231	411	1	C->T	CGG->TGG	R->W
	1009	337	1	C->T	CCA->TCA	P->S
	**406**	**136**	**1**	**C->T**	**CGT->TGT**	**R->C**
	**391**	**131**	**1**	**C->T**	**CGT->TGT**	**R->C**
	334	112	1	C->T	CTT->TTT	L->F
	310	104	1	C->T	CGT->TGT	R->C
	303	101	3	C->T	GCC->GCT	A->A
	**155**	**52**	**2**	**C->T**	**TCA->TTA**	**S->L**
	**151**	**51**	**1**	**C->T**	**CCT->TCT**	**P->S**
	**122**	**41**	**2**	**C->T**	**TCT->TTT**	**S->F**
	**119**	**40**	**2**	**C->T**	**TCT->TTT**	**S->F**
	52	18	1	C->T	CGT->TGT	R->C
	50	17	2	C->T	CCT->CTT	P->L
	38	13	2	C->T	TCC->TTC	S->F
	35	12	2	C->T	ACT->ATT	T->I
ccmFN	**1507**	**503**	**1**	**C->T**	**CCC->TCC**	**P->S**
	**1472**	**491**	**2**	**C->T**	**TCA->TTA**	**S->L**
	**1460**	**487**	**2**	**C->T**	**CCA->CTA**	**P->L**
	**1456**	**486**	**1**	**C->T**	**CTT->TTT**	**L->F**
	**1436**	**479**	**2**	**C->T**	**TCG->TTG**	**S->L**
	**1417**	**473**	**1**	**C->T**	**CTT->TTT**	**L->F**
	**1375**	**459**	**1**	**C->T**	**CGG->TGG**	**R->W**
	**1342**	**448**	**1**	**C->T**	**CGG->TGG**	**R->W**
	**1324**	**442**	**1**	**C->T**	**CGG->TGG**	**R->W**
	**1309**	**437**	**1**	**C->T**	**CAT->TAT**	**H->Y**
	**1292**	**431**	**2**	**C->T**	**CCA->CTA**	**P->L**
	**1264**	**422**	**1**	**C->T**	**CGG->TGG**	**R->W**
	**973**	**325**	**1**	**C->T**	**CGT->TGT**	**R->C**
	**800**	**267**	**2**	**C->T**	**TCA->TTA**	**S->L**
	**785**	**262**	**2**	**C->T**	**CCA->CTA**	**P->L**
	**773**	**258**	**2**	**C->T**	**TCA->TTA**	**S->L**
	**751**	**251**	**1**	**C->T**	**CGT->TGT**	**R->C**
	**713**	**238**	**2**	**C->T**	**TCG->TTG**	**S->L**
	704	235	2	C->T	CCT->CTT	P->L
	604	202	1	C->T	CAT->TAT	H->Y

	378	126	3	C->T	TTC->TTT	F->F
	**371**	**124**	**2**	**C->T**	**TCG->TTG**	**S->L**
	**289**	**97**	**1**	**C->T**	**CTT->TTT**	**L->F**
	**262**	**88**	**1**	**C->T**	**CGG->TGG**	**R->W**
	**254**	**85**	**2**	**C->T**	**TCA->TTA**	**S->L**
	**200**	**67**	**2**	**C->T**	**TTC->TTT**	**P→L**
	**157**	**53**	**1**	**C->T**	**CCT->TCT**	**P->S**
	**148**	**50**	**1**	**C->T**	**CGT->TGT**	**R->C**
	**143**	**48**	**2**	**C->T**	**CCG->CTG**	**P->L**
	**104**	**35**	**2**	**C->T**	**CCT->CTT**	**P->L**
	**44**	**15**	**2**	**C->T**	**CCG->CTG**	**P->L**
cob	229	77	1	C->T	CCG->TCG	P->S
	**397**	**133**	**1**	**C->T**	**CTC->TTC**	**L->F**
	**436**	**146**	**1**	**C->T**	**CAT->TAT**	**H->Y**
	**469**	**157**	**1**	**C->T**	**CGG->TGG**	**R->W**
	**530**	**177**	**2**	**C->T**	**CCA->CTA**	**P->L**
	**679**	**227**	**1**	**C->T**	**CAT->TAT**	**H->Y**
	**791**	**264**	**2**	**C->T**	**TCT->TTT**	**S->F**
	**848**	**283**	**2**	**C->T**	**TCT->TTT**	**S->F**
	**919**	**307**	**1**	**C->T**	**CCC->TCC**	**P->S**
	**964**	**322**	**1**	**C->T**	**CAT->TAT**	**H->Y**
	**1019**	**340**	**2**	**C->T**	**CCA->CTA**	**P->L**
	**1093**	**365**	**1**	**C->T**	**CAC->TAC**	**H->Y**
	**1126**	**376**	**1**	**C->T**	**CGC->TGC**	**R->C**
	**1195**	**399**	**1**	**C->T**	**CCT->TCT**	**P->S**
cox1	**122**	**41**	**2**	**C->T**	**CCG->CTG**	**P->L**
	126	42	3	C->T	GTC->GTT	V->V
	**353**	**118**	**2**	**C->T**	**TCT->TTT**	**S->F**
	**365**	**122**	**2**	**C->T**	**TCT->TTT**	**S->F**
	**463**	**155**	**1**	**C->T**	**CCA->TCA**	**P->S**
	**554**	**185**	**2**	**C->T**	**TCA->TTA**	**S->L**
	**563**	**188**	**2**	**C->T**	**TCT->TTT**	**S->F**
	**569**	**190**	**2**	**C->T**	**CCT->CTT**	**P->L**
	**626**	**209**	**2**	**C->T**	**TCC->TTC**	**S->F**
	**662**	**221**	**2**	**C->T**	**TCA->TTA**	**S->L**
	**701**	**234**	**2**	**C->T**	**CCA->CTA**	**P->L**
	**779**	**260**	**2**	**C->T**	**TCT->TTT**	**S->F**
	**826**	**276**	**1**	**C->T**	**CGG->TGG**	**R->W**
	**857**	**286**	**2**	**C->T**	**CCC->CTC**	**P->L**
	**872**	**291**	**2**	**C->T**	**TCC->TTC**	**S->F**
	**965**	**322**	**2**	**C->T**	**CCT->CTT**	**P->L**
	**971**	**324**	**2**	**C->T**	**TCT->TTT**	**S->F**
	**1148**	**383**	**2**	**C->T**	**TCC->TTC**	**S->F**
	**1190**	**397**	**2**	**C->T**	**CCG->CTG**	**P->L**
	**1297**	**433**	**1**	**C->T**	**CAC->TAC**	**H->Y**
	**1385**	**462**	**2**	**C->T**	**CCG->CTG**	**P->L**
	1398	466	3	C->T	CCC->CCT	P->P
	**1516**	**506**	**1**	**C->T**	**CGT->TGT**	**R->C**
	**1544**	**515**	**2**	**C->T**	**TCA->TTA**	**S->L**
	**1600**	**534**	**1**	**C->T**	**CCA->TCA**	**P->S**

	**1610**	**537**	**2**	**C->T**	**CCG->CTG**	**P->L**
cox2	**71**	**24**	**2**	**C->T**	**TCT->TTT**	**S->F**
	**161**	**54**	**2**	**C->T**	**TCA->TTA**	**S->L**
	**163**	**55**	**1**	**C->T**	**CGG->TGG**	**R->W**
	**253**	**85**	**1**	**C->T**	**CGG->TGG**	**R->W**
	**278**	**93**	**2**	**C->T**	**CCG->CTG**	**P->L**
	379	127	1	C->T	CGG->TGG	R->W
	**488**	**163**	**2**	**C->T**	**ACG->ATG**	**T->M**
	**626**	**209**	**2**	**C->T**	**TCA->TTA**	**S->L**
	**668**	**223**	**2**	**C->T**	**ACC->ATC**	**T->I**
	**677**	**226**	**2**	**C->T**	**TCG->TTG**	**S->L**
	**766**	**256**	**1**	**C->T**	**TCG->TTG**	**S->L**
	**781**	**263**	**1**	**C->T**	**TCG->TTG**	**S->L**
cox3	**245**	**82**	**2**	**C->T**	**CCT->CTT**	**P->L**
	**289**	**97**	**1**	**C->T**	**CTT->TTT**	**L->F**
	**298**	**100**	**1**	**C->T**	**CTT->TTT**	**L->F**
	**304**	**102**	**1**	**C->T**	**CGG->TGG**	**R->W**
	**311**	**104**	**2**	**C->T**	**TCT->TTT**	**S->F**
	**314**	**105**	**2**	**C->T**	**TCT->TTT**	**S->F**
	**388**	**130**	**1**	**C->T**	**CGG->TGG**	**R->W**
	**419**	**140**	**2**	**C->T**	**CCC->CTC**	**P->L**
	**512**	**171**	**2**	**C->T**	**TCA->TTA**	**S->L**
	**566**	**189**	**2**	**C->T**	**TCC->TTC**	**S->F**
	**653**	**218**	**2**	**C->T**	**TCG->TTG**	**S->L**
	**754**	**252**	**1**	**C->T**	**CGG->TGG**	**R->W**
	**764**	**255**	**2**	**C->T**	**CCA->CTA**	**P->L**
matR	**1844**	**615**	**2**	**C->T**	**TCA->TTA**	**S->L**
	**1826**	**609**	**2**	**C->T**	**CCA->CTA**	**P->L**
	**1787**	**596**	**2**	**C->T**	**CCG->CTG**	**P->L**
	**1756**	**586**	**1**	**C->T**	**CAC->TAC**	**H->Y**
	**1720**	**574**	**1**	**C->T**	**CGC->TGC**	**R->C**
	**1700**	**567**	**2**	**C->T**	**CCT->CTT**	**P->L**
	**1679**	**560**	**2**	**C->T**	**TCC->TTC**	**S->F**
	1545	515	3	C->T	CCC->CCT	P->P
	413	138	2	C->T	TCG->TTG	S->L
	**326**	**109**	**2**	**C->T**	**CCA->CTA**	**P->L**
	**236**	**79**	**2**	**C->T**	**TCC->TTC**	**S->F**
	193	65	1	C->T	CCC->TCC	P->S
	147	49	3	C->T	TTC->TTT	F->F
	43	15	1	C->T	CCC->TCC	P->S
	**32**	**11**	**2**	**C->T**	**TCC->TTC**	**S->F**
mttB	**23**	**8**	**2**	**C->T**	**CCG->CTG**	**P->L**
	56	19	2	C->T	TCC->TTC	S->F
	61	21	1	C->T	CGG->TGG	R->W
	**97**	**33**	**1**	**C->T**	**CGT->TGT**	**R->C**
	**109**	**37**	**1**	**C->T**	**CCG->TCG**	**P->S**
	**125**	**42**	**2**	**C->T**	**TCT->TTT**	**S->F**
	**128**	**43**	**2**	**C->T**	**CCA->CTA**	**P->L**
	**181**	**61**	**1**	**C->T**	**CGT->TGT**	**R->C**
	**191**	**64**	**2**	**C->T**	**TCA->TTA**	**S->L**

	**203**	**68**	**2**	**C->T**	**TCC->TTC**	**S->F**
	**205**	**69**	**1**	**C->T**	**CCG->TCG**	**P->S**
	255	85	3	C->T	CCC->CCT	P->P
	**265**	**89**	**1**	**C->T**	**CAT->TAT**	**H->Y**
	**334**	**112**	**1**	**C->T**	**CAT->TAT**	**H->Y**
	349	117	1	C->T	CGC->TGC	R->C
	**356**	**119**	**2**	**C->T**	**TCC->TTC**	**S->F**
	**379**	**127**	**1**	**C->T**	**CCC->TCC**	**P->S**
	**410**	**137**	**2**	**C->T**	**CCA->CTA**	**P->L**
	450	150	3	C->T	ATC->ATT	I->I
	**475**	**159**	**1**	**C->T**	**CAT->TAT**	**H->Y**
	500	167	2	C->T	TCG->TTG	S->L
	**508**	**170**	**1**	**C->T**	**CCA->TCA**	**P->S**
	**544**	**182**	**1**	**C->T**	**CGT->TGT**	**R->C**
	**551**	**184**	**2**	**C->T**	**CCA->CTA**	**P->L**
	**557**	**186**	**2**	**C->T**	**CCA->CTA**	**P->L**
	**613**	**205**	**1**	**C->T**	**CCG->TCG**	**P->S**
	619	207	1	C->T	CTC->TTC	L->F
	670	224	1	C->T	CCT->TCT	P->S
	**707**	**236**	**2**	**C->T**	**TCT->TTT**	**S->F**
	**716**	**239**	**2**	**C->T**	**TCG->TTG**	**S->L**
nad1	**215**	**72**	**2**	**C->T**	**TCC->TTC**	**S->F**
	**265**	**89**	**1**	**C->T**	**CGG->TGG**	**R->W**
	**308**	**103**	**2**	**C->T**	**CCG->CTG**	**P->L**
	**376**	**126**	**1**	**C->T**	**CGG->TGG**	**R->W**
	**436**	**146**	**1**	**C->T**	**CCT->TCT**	**P->S**
	**490**	**164**	**1**	**C->T**	**CCC->UCC**	**P->S**
	**493**	**165**	**1**	**C->T**	**CGT->TGT**	**R->C**
	**500**	**167**	**2**	**C->T**	**TCG->TTG**	**S->L**
	**536**	**179**	**2**	**C→T**	**TCC->TTC**	**S->F**
	**635**	**212**	**2**	**C->T**	**TCA->TTA**	**S->L**
	**937**	**313**	**1**	**C->T**	**CCC->TCC**	**P->S**
	**928**	**310**	**1**	**C->T**	**CGG->TGG**	**R->W**
	**898**	**300**	**1**	**C->T**	**CGG->TGG**	**R->W**
	**802**	**268**	**1**	**C->T**	**CGG->TGG**	**R->W**
	789	263	3	C->T	ATC->ATT	I->I
	779	260	2	C->T	TCA->TTA	S->L
	**755**	**252**	**2**	**C->T**	**CCG->CTG**	**P->L**
nad2	**1285**	**429**	**1**	**C->T**	**CGT->TGT**	**R->C**
	1255	419	1	C->T	CCA->TCA	P->S
	**1136**	**379**	**2**	**C->T**	**TCG->TTG**	**S->L**
	**1067**	**356**	**2**	**C->T**	**TCA->TTA**	**S->L**
	**1037**	**346**	**2**	**C->T**	**TCA->TTA**	**S->L**
	**967**	**323**	**1**	**C->T**	**CGT->TGT**	**R->C**
	**937**	**313**	**1**	**C->T**	**CAT->TAT**	**H->Y**
	**818**	**273**	**2**	**C->T**	**TCT->TTT**	**S->F**
	**809**	**270**	**2**	**C->T**	**TCA->TTA**	**S->L**
	**797**	**266**	**2**	**C->T**	**TCT->TTT**	**S->F**
	**686**	**229**	**2**	**C->T**	**TCC->TTC**	**S->F**
	**671**	**224**	**2**	**C->T**	**TCT->TTT**	**S->F**

	**26**	**9**	**2**	**C->T**	**TCC->TTC**	**S->F**
	232	78	1	C->T	CTT->TTT	L->F
	**317**	**106**	**2**	**C->T**	**TCT->TTT**	**S->F**
	**320**	**107**	**2**	**C->T**	**TCC->TTC**	**S->F**
	**344**	**115**	**2**	**C->T**	**TCT->TTT**	**S->F**
	**350**	**117**	**2**	**C->T**	**TCC->TTC**	**S->F**
	**376**	**126**	**1**	**C->T**	**CGC->TGC**	**R->C**
	**410**	**137**	**2**	**C->T**	**TCA->TTA**	**S->L**
	**437**	**146**	**2**	**C->T**	**CCT->CTT**	**P->L**
	**506**	**169**	**2**	**C->T**	**TCG->TTG**	**S->L**
nad3	**5**	**2**	**2**	**C->T**	**TCA->TTA**	**S->L**
	**44**	**15**	**2**	**C->T**	**CCG->CTG**	**P->L**
	**62**	**21**	**2**	**C->T**	**CCA->CTA**	**P->L**
	**80**	**27**	**2**	**C->T**	**CCA->CTA**	**P->L**
	**208**	**70**	**1**	**C->T**	**CCT->TTT**	**P->L**
	**209**	**70**	**2**	**C->T**	**CCT->TTT**	**P->L**
	**215**	**72**	**2**	**C->T**	**CCG->CTG**	**P->L**
	**247**	**83**	**1**	**C->T**	**CCT->TCT**	**P->S**
	**275**	**92**	**2**	**C->T**	**TCT->TTT**	**S->F**
	**317**	**106**	**2**	**C->T**	**TCT->TTT**	**S->F**
	**344**	**115**	**2**	**C->T**	**TCG->TTG**	**S->L**
	**349**	**117**	**1**	**C->T**	**CGG->TGG**	**R->W**
nad4	**1430**	**477**	**2**	**C->T**	**TCG->TTG**	**S->L**
	**433**	**145**	**1**	**C->T**	**CTT->TTT**	**L->F**
	**416**	**139**	**2**	**C->T**	**CCT->CTT**	**P->L**
	**403**	**135**	**1**	**C->T**	**CGC->TGC**	**R->C**
	**376**	**126**	**1**	**C->T**	**CGT->TGT**	**R->C**
	**368**	**123**	**2**	**C->T**	**TCT->TTT**	**S->F**
	**362**	**121**	**2**	**C->T**	**ACA->ATA**	**T->I**
	261	87	3	C->T	GAC->GAT	D->D
	**197**	**66**	**2**	**C->T**	**TCT->TTT**	**S->F**
	**166**	**56**	**1**	**C->T**	**CGG->TGG**	**R->W**
	**158**	**53**	**2**	**C->T**	**CCT->CTT**	**P->L**
	**107**	**36**	**2**	**C->T**	**CCG->CTG**	**P->L**
	**77**	**26**	**2**	**C->T**	**CCT->CTT**	**P->L**
	**74**	**25**	**2**	**C->T**	**ACT->ATT**	**T->I**
	**29**	**10**	**2**	**C->T**	**TCT->TTT**	**S->F**
nad4L	**251**	**84**	**2**	**C->T**	**TCT->TTT**	**S->F**
	**167**	**56**	**2**	**C->T**	**CCA->CTA**	**P->L**
	**158**	**53**	**2**	**C->T**	**TCA->TTA**	**S->L**
	149	50	2	C->T	TCA->TTA	S->L
	**128**	**43**	**2**	**C->T**	**TCG->TTG**	**S->L**
	101	34	2	C->T	TCG->TTG	S->L
	**80**	**27**	**2**	**C->T**	**TCA->TTA**	**S->L**
	**56**	**19**	**2**	**C->T**	**CCT->CTT**	**P->L**
	**25**	**9**	**1**	**C->T**	**CGG->TGG**	**R->W**
	**17**	**6**	**2**	**C->T**	**TCA->TTA**	**S->L**
	**11**	**4**	**2**	**C->T**	**TCT->TTT**	**S->F**
nad5	1318	440	1	C->T	CTA->TTA	L->L
	**1310**	**437**	**2**	**C->T**	**TCA->TTA**	**S->L**

	**1184**	**395**	**2**	**C->T**	**CCA->CTA**	**P->L**
	**835**	**279**	**1**	**C->T**	**CCA->TCA**	**P->S**
	**725**	**242**	**2**	**C->T**	**TCA->TTA**	**S->L**
	**713**	**238**	**2**	**C->T**	**TCG->TTG**	**S->L**
	**689**	**230**	**2**	**C->T**	**GCT->GTT**	**A->V**
	**676**	**226**	**1**	**C->T**	**CTT->TTT**	**L->F**
	**631**	**211**	**1**	**C->T**	**CGC->TGC**	**R->C**
	**629**	**210**	**2**	**C->T**	**TCT->TTT**	**S->F**
	**548**	**183**	**2**	**C->T**	**TCG->TTG**	**S->L**
	**539**	**180**	**2**	**C->T**	**CCT->CTT**	**P->L**
	**398**	**133**	**2**	**C->T**	**TCT->TTT**	**S->F**
	**374**	**125**	**2**	**C->T**	**CCA->CTA**	**P->L**
	**359**	**120**	**2**	**C->T**	**CCT->TTT**	**P->F**
	**358**	**120**	**1**	**C->T**	**CCT->TTT**	**P->F**
	272	91	2	C->T	TCC->TTC	S->F
	**242**	**81**	**2**	**C->T**	**CCG->CTG**	**P->L**
	**155**	**52**	**2**	**C->T**	**CCG->CTG**	**P->L**
	**1982**	**661**	**2**	**C->T**	**TCG->TTG**	**S->L**
	**1942**	**648**	**2**	**C->T**	**CGT->TGT**	**R->C**
	**1940**	**647**	**1**	**C->T**	**TCT->TTT**	**S->F**
	**1919**	**640**	**2**	**C->T**	**TCA->TTA**	**S->L**
	1586	529	2	C->T	CCA->CTA	P->L
	**1565**	**522**	**2**	**C->T**	**TCT->TTT**	**S->F**
	**1556**	**519**	**2**	**C->T**	**TCA->TTA**	**S->L**
	**1526**	**509**	**2**	**C->T**	**ACC->ATC**	**T->I**
	**1466**	**489**	**2**	**C->T**	**CCC->CTC**	**P->L**
nad6	**569**	**190**	**2**	**C->T**	**TCT->TTT**	**S->F**
	463	155	1	C->T	CCT->TCT	P->S
	**446**	**149**	**2**	**C->T**	**TCC->TTC**	**S->F**
	**191**	**64**	**2**	**C->T**	**TCA->TTA**	**S->L**
	**169**	**57**	**1**	**C->T**	**CAT->TAT**	**H->Y**
	**161**	**54**	**2**	**C->T**	**CCA->CTA**	**P->L**
	**103**	**35**	**1**	**C->T**	**CGC->TGC**	**R->C**
	**95**	**32**	**2**	**C->T**	**CCA->CTA**	**P->L**
	**89**	**30**	**2**	**C->T**	**CCC->TTC**	**P->F**
	**88**	**30**	**1**	**C->T**	**CCC->TTC**	**P->F**
nad7	**1154**	**385**	**2**	**C->T**	**TCT->TTT**	**S->F**
	**1112**	**371**	**2**	**C->T**	**CCA->CTA**	**P->L**
	**1091**	**364**	**2**	**C->T**	**TCT->TTT**	**S->F**
	**1076**	**359**	**2**	**C->T**	**TCA->TTA**	**S->L**
	**1067**	**356**	**2**	**C->T**	**TCT->TTT**	**S->F**
	**1045**	**349**	**1**	**C->T**	**CGT->TGT**	**R->C**
	1038	346	3	C->T	CCC->CCT	P->P
	**932**	**311**	**2**	**C->T**	**CCT->CTT**	**P->L**
	**764**	**255**	**2**	**C->T**	**CCT->CTT**	**P->L**
	**697**	**233**	**1**	**C->T**	**CGC->TGC**	**R->C**
	**668**	**223**	**2**	**C->T**	**CCT->TTT**	**P->F**
	**667**	**223**	**1**	**C->T**	**CCT->TTT**	**P->F**
	**662**	**221**	**2**	**C->T**	**TCG->TTG**	**S->L**
	**652**	**218**	**1**	**C->T**	**CAT->TAT**	**H->Y**

	**626**	**209**	**2**	**C->T**	**TCG->TTG**	**S->L**
	**512**	**171**	**2**	**C->T**	**TCA->TTA**	**S->L**
	**467**	**156**	**2**	**C->T**	**TCC->TTC**	**S->F**
	**317**	**106**	**2**	**C->T**	**TCA->TTA**	**S->L**
	**278**	**93**	**2**	**C->T**	**TCA->TTA**	**S->L**
	**269**	**90**	**2**	**C->T**	**TCA->TTA**	**S->L**
	**250**	**84**	**1**	**C->T**	**CGT->TGT**	**R->C**
	**185**	**62**	**2**	**C->T**	**TCA->TTA**	**S->L**
	**178**	**60**	**1**	**C->T**	**CAT->TAT**	**H->Y**
	**137**	**46**	**2**	**C->T**	**TCA->TTA**	**S->L**
	**83**	**28**	**2**	**C->T**	**TCA->TTA**	**S->L**
	**77**	**26**	**2**	**C->T**	**TCA->TTA**	**S->L**
	45	15	3	C->T	TTC->TTT	F->F
	**38**	**13**	**2**	**C->T**	**TCG->TTG**	**S->L**
nad9	**92**	**31**	**2**	**C->T**	**TCT->TTT**	**S->F**
	**113**	**38**	**2**	**C->T**	**CCA->CTA**	**P->L**
	**167**	**56**	**2**	**C->T**	**TCG->TTG**	**S->L**
	**190**	**64**	**1**	**C->T**	**CAT->TAT**	**H->Y**
	**298**	**100**	**1**	**C->T**	**CCG->TCG**	**P->S**
	**311**	**104**	**2**	**C->T**	**CCA->CTA**	**P->L**
	**328**	**110**	**1**	**C->T**	**CGG->TGG**	**R->W**
	**368**	**123**	**2**	**C->T**	**TCC->TTC**	**S->F**
	**398**	**133**	**2**	**C->T**	**TCA->TTA**	**S->L**
	**439**	**147**	**1**	**C->T**	**CTT->TTT**	**L->F**
	**539**	**180**	**2**	**C->T**	**TCT->TTT**	**S->F**
rpl10	239	80	2	C->T	TCG->TTG	S->L
	180	60	3	C->T	ACC->ACT	T->T
	134	45	2	C->T	CCA->CTA	P->L
	101	34	2	C->T	TCA->TTA	S->L
	83	28	2	C->T	TCA->TTA	S->L
rpl16	**185**	**62**	**2**	**C->T**	**ACT->ATT**	**T->I**
	**313**	**105**	**1**	**C->T**	**TCA->TTA**	**L->F**
	**488**	**163**	**2**	**C->T**	**TCA->TTA**	**S->L**
rpl5	**521**	**174**	**2**	**C->T**	**CCG->CTG**	**P->L**
	**518**	**173**	**2**	**C->T**	**CCA->CTA**	**P->L**
	169	57	1	C->T	CCG->TCG	P->S
	**92**	**31**	**2**	**C->T**	**TCG->TTG**	**S->L**
	**59**	**20**	**2**	**C->T**	**CCG->CTG**	**P->L**
	**47**	**16**	**2**	**C->T**	**CCG->CTG**	**P->L**
	**35**	**12**	**2**	**C->T**	**TCA->TTA**	**S->L**
rps12	**71**	**24**	**2**	**C->T**	**TCG->TTG**	**S->L**
	**100**	**34**	**1**	**C->T**	**CGC->TGC**	**R->C**
	**104**	**35**	**2**	**C->T**	**CCG->CTG**	**P->L**
	**112**	**38**	**1**	**C->T**	**CCA->TCA**	**P->S**
	**146**	**49**	**2**	**C->T**	**CCA->CTA**	**P->L**
	**196**	**66**	**1**	**C->T**	**CAC->TAC**	**H->Y**
	**221**	**74**	**2**	**C->T**	**TCG->TTG**	**S->L**
	**269**	**90**	**2**	**C->T**	**TCG->TTG**	**S->L**
	**284**	**95**	**2**	**C->T**	**TCC->TTC**	**S->F**
rps13	26	9	2	C->T	TCA->TTA	S->L

	56	19	2	C->T	TCA->TTA	S->L
	100	34	1	C->T	CGT->TGT	R->C
	287	96	2	C->T	TCA->TTA	S->L
rps3	86	29	2	C->T	TCA->TTA	S->L
	120	40	3	C->T	TTC->TTT	F->F
	**506**	**169**	**2**	**C->T**	**TCA->TTA**	**S->L**
	**710**	**237**	**2**	**C->T**	**TCG->TTG**	**S->L**
	**1019**	**340**	**2**	**C->T**	**CCA->CTA**	**P->L**
	**1349**	**450**	**2**	**C->T**	**CCG->CTG**	**P->L**
	**1376**	**459**	**2**	**C->T**	**CCG->CTG**	**P->L**
	1487	496	2	C->T	TCA->TTA	S->L
	1573	525	1	C->T	CCT->TCT	P->S
	**1604**	**535**	**2**	**C->T**	**TCA->TTA**	**S->L**
rps4	**184**	**62**	**1**	**C->T**	**CCC->TCC**	**P->S**
	**193**	**65**	**1**	**C->T**	**CAT->TAT**	**H->Y**
	**257**	**86**	**2**	**C->T**	**CCA->CTA**	**P->L**
	**266**	**89**	**2**	**C->T**	**CCA->CTA**	**P->L**
	278	93	2	C->T	TCG->TTG	S->L
	290	97	2	C->T	CCG->CTG	P->L
	**335**	**112**	**2**	**C->T**	**CCG->CTG**	**P->L**
	461	154	2	C->T	TCC->TTC	S->F
	**479**	**160**	**2**	**C->T**	**TCA->TTA**	**S->L**
	**905**	**302**	**2**	**C->T**	**TCG->TTG**	**S->L**
	**916**	**306**	**1**	**C->T**	**CAT->TAT**	**H->Y**
	926	309	2	C->T	CCA->CTA	P->L
	**941**	**314**	**2**	**C->T**	**TCT->TTT**	**S->F**

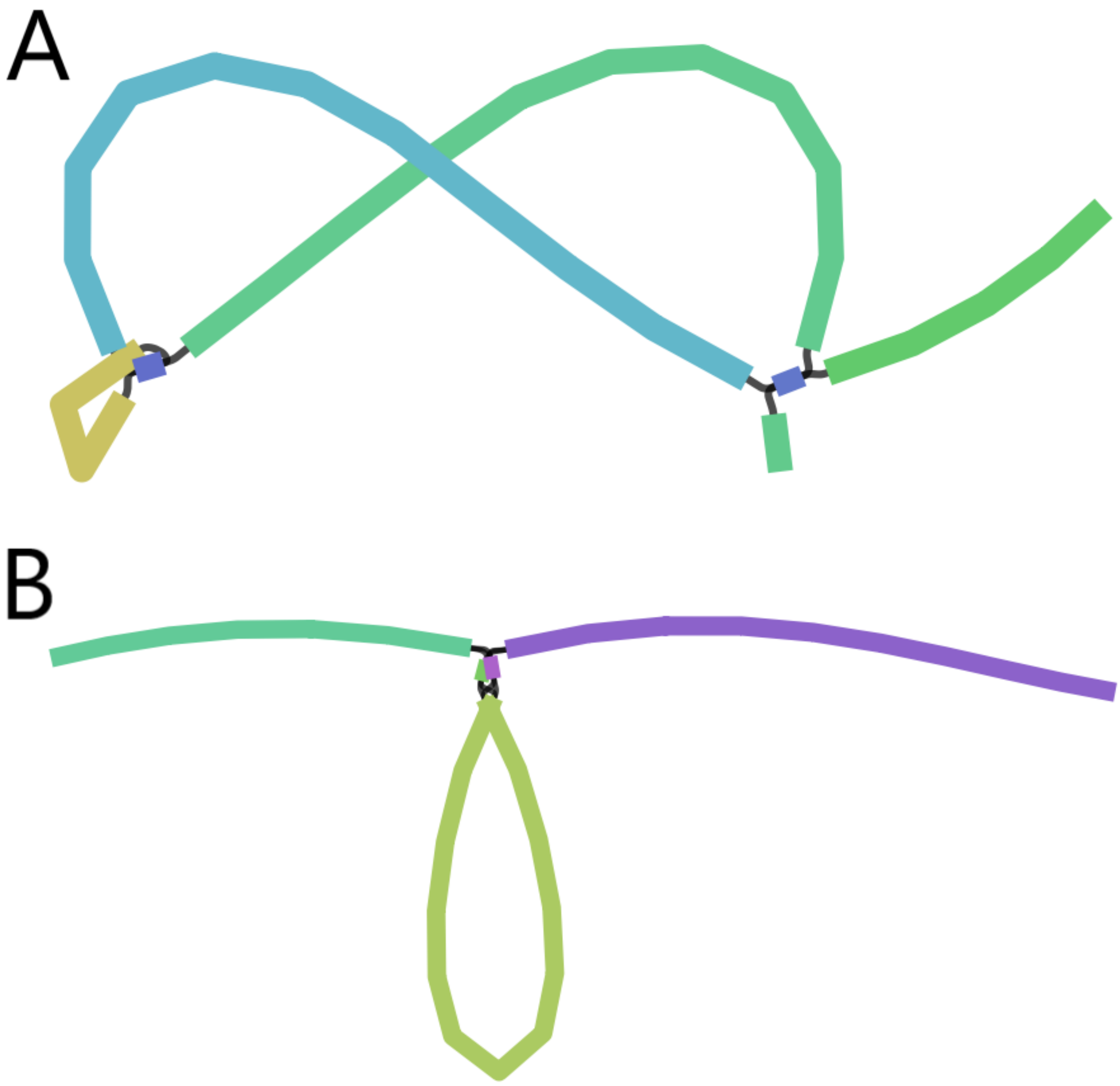

Figure S1. Manually curated final assembly graphs of A) *H. argophyllus* and B) *H. annuus* VIR114A x *H. argophyllus* hybrid visualized using Bandage software. All the contigs displayed are part of the mitochondrial master circle, but the links between some contigs are absent in the graphs (low-coverage regions, which were checked by PCR and reads remapping). The displayed circles are the sub-circle structures. Such sub-circle structures can be resolved in the final graph, which will be look like one straight line.

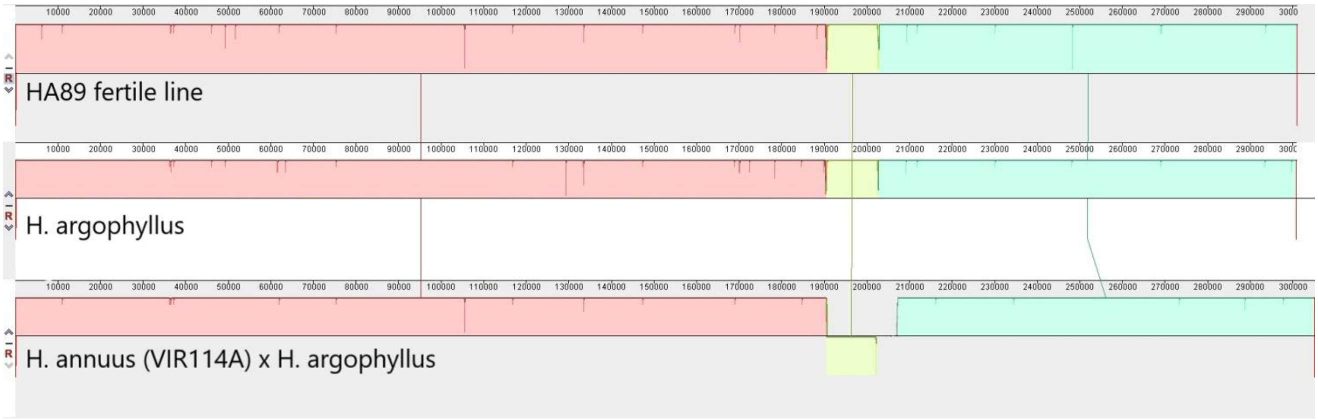

Figure S2. The progressive MAUVE alignment of complete mitogenomes: *H. annuus* fertile line HA89 (MG735191.1 NCBI accession), *H. argophyllus* and the hybrid (*H. annuus* VIR114A x *H. argophyllus)*

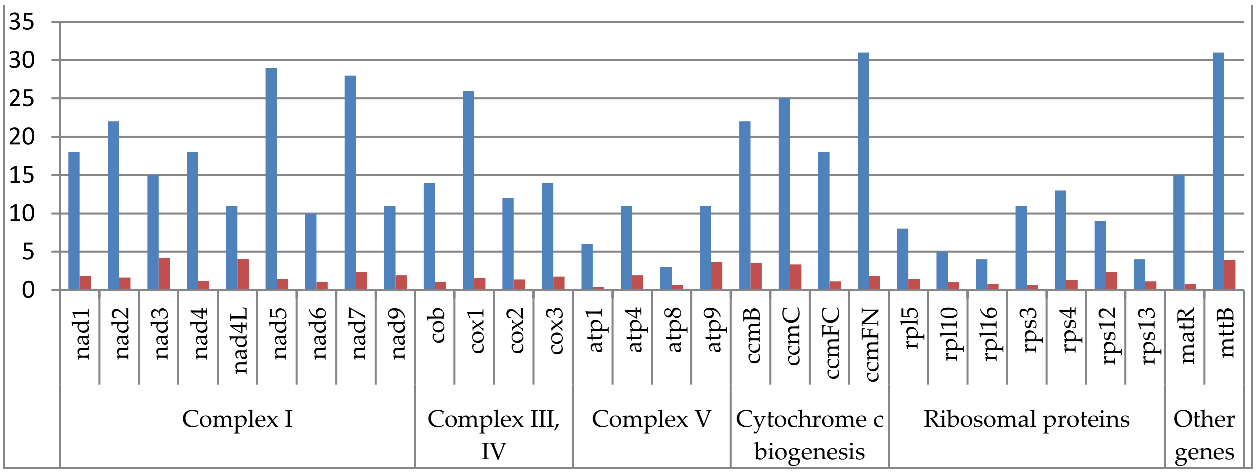

Figure S3. The absolute number of RNA editing substitutions per gene (blue bars) and the relative number of RNA editing substitutions by gene length normalized to 100 bp (red bars) revealed by RNAseq reads mapping
